# Mechanistic Insights into Immune Suppression and Evasion in Bacterial Vaginosis

**DOI:** 10.1007/s00284-022-02771-2

**Published:** 2022-02-07

**Authors:** Emmanuel Amabebe, Dilly O. C. Anumba

**Affiliations:** Academic Unit of Reproductive and Developmental Medicine-Obstetrics and Gynaecology, Department of Oncology and Metabolism, The University of Sheffield, 4th Floor, Jessop Wing, Tree Root Walk, Sheffield, S10 2SF UK

## Abstract

The immunological response to bacterial vaginosis (BV) remains poorly understood and recurrent BV is still a major public health burden especially in the pregnant population. This article reviews the potential mechanisms by which BV-associated bacteria suppress and circumvent the host and microbial defence responses, and propagate their survival/dominance without overt inflammation. We discuss the composition of cervicovaginal mucosal barrier and the mechanism by which BV circumvents host defence: the degradation of the mucosal barrier and immunoglobulin A (IgA); the BV-associated organism *Gardnerella vaginalis* haemolysin (vaginolysin); diminished IgA response against vaginolysin; mucosal sialic acid degradation, foraging and depletion; inhibition of IL-8-induced neutrophilic infiltration; and metabolite-induced incapacitation of neutrophil and monocyte chemotaxis. We also highlight the tolerance/resistance to both host and antimicrobial molecules mounted by BV-associated biofilms. A plausible role of sialic acid-binding immunoglobulin-like lectins (SIGLECS) was also suggested. Sialidase, which is often produced by *G. vaginalis*, is central to the immunosuppression, relapse and recurrence observed in BV, although it is supported by other hydrolytic enzymes, vaginolysin and immunomodulatory metabolites.

## Introduction

Bacterial vaginosis (BV) is a polymicrobial disorder of the lower genital tract characterised by an alteration in the vaginal microenvironment (dysbiosis) resulting in the loss of *Lactobacillus* species dominance, increase in vaginal pH and a dramatic overgrowth of pathogenic Gram negative and positive facultative and obligate anaerobic bacteria such as *Gardnerella, Atopobium, Bacteroides, Mobiluncus, Prevotella, Mycoplasma, Peptostreptococcus, Anaerococcus, Sneathia, Clostridium, Leptotrichia* species, BV-associated bacterium 1 (BVAB1) to BVAB3 etcetera [1–7]. The specific vaginal bacterial composition of BV can differ between individual women [5, 8, 9]. However, one frequent culprit in the pathogenesis and diagnosis of BV is *Gardnerella vaginalis*, a non-motile, catalase-negative, Gram variable facultative anaerobic coccobacilli [6, 7]. Although *G. vaginalis* was initially regarded as the sole cause of BV-related clinical signs and symptoms [10], i.e. “primary pathogen model” [7], synergistic contributions from other anaerobic pathogens have been reported more recently [11–13]. Despite its pathogenic potential, *G. vaginalis* is present in the vagina of most women (including > 60% without BV), although women with BV have ~ fourfold higher levels compared to women without BV [14, 15]. Unlike the strict anaerobes such as *Prevotella* spp., *G. vaginalis* can adhere to the vaginal epithelium at pH of 4–5 and tolerate environments with high redox potential [7]. The BV-associated microbiota has been studied and reported extensively [1, 8, 16, 17] and is beyond the scope of the current report. However, the mechanisms (e.g. sialidase and metabolite activities) employed by *G. vaginalis* and other anaerobes to contribute to the features and health complications associated with BV [6] are discussed in this review.

The dysbiotic vaginal econiche in BV can be induced by several factors including hormonal changes, menstruation, pregnancy, multiple sex partners, smoking, poor personal hygiene, use of contraceptives, antibiotic therapy, socioeconomic status, psychosocial stress, and some infections and disorders such as diabetes mellitus or insulin resistance [1, 4, 18].

BV is the most common vaginal disorder of reproductive-age women worldwide [3] including premenopausal, fertile and pregnant women [4], with an annual estimated treatment cost of $4.8 billion [19]. The economic burden of BV can triple when the cost of BV-associated preterm birth and human immunodeficiency virus (HIV) cases are included [19]. The global prevalence of BV is presented in Table 1[19], and prevalence rates range from 5 to 70% [1, 17]. BV is a major public health burden as it is associated with poor reproductive outcomes including preterm birth, low birth weight, chorioamnionitis, amniotic fluid infection, preterm rupture of membranes, miscarriage, failure of in vitro fertilisation, pelvic inflammatory disease, postpartum endometritis and increased risk of acquisition and transmission of HIV and other sexually transmitted infections (STIs) [1, 5, 20–23].

**Table 1:** Global prevalence of bacterial vaginosis [19]

Region	Prevalence (%)
South Asia	29
North America	27
Middle East and North Africa	25
Sub-Saharan Africa	25
East Asia and Pacific	24
Latin America and Caribbean	24
Europe and Central Asia	23

There is also considerable racial/ethnic variance in bacterial vaginosis (BV) prevalence. In both general and pregnant population, prevalence of BV is higher in blacks compared with other racial and ethnic groups. Within North America, prevalence of BV in the general population was significantly higher in blacks (33%) and Hispanic women (31%), than white (23%) and Asian (11%) women [19]. Such disparities may be influenced by socioeconomic factors and biological factors such as lower concentrations of health-promoting vaginal *Lactobacillus* spp. in black women [35]. The mechanisms underpinning the racial/ethnic differences in the prevalence of BV are crucial and warrants further investigation. However, this is beyond the scope of this review

BV is usually asymptomatic. However, in severe cases symptoms such as vaginal discomfort, and a non-itchy fishy or malodourous homogeneous creamy/greyish vaginal discharge [1, 4, 19] that may be more noticeable during menstruation or after sexual intercourse have been reported [1]. Many women with BV only complain of malodorous vaginal discharge without an overt inflammation leading to the term “vaginosis” instead of “vaginitis”, which is an inflammation of the vagina [24].

BV is an enigmatic syndrome with controversial aetiology [20]. A decline in the health-promoting *Lactobacillus* species leads to a decrease in lactic acid that acidifies the vaginal milieu. The increased pH of the ecosystem creates a conducive environment for the proliferation of mixed anaerobes that were hitherto kept dormant by lactobacilli and their antimicrobial by-products including lactic acid, H_2_O_2_, bacteriocins and biosurfactants [1]. The resultant heterogeneous vaginal space with pH > 4.5, increased bacterial load and species diversity also has increased concentrations of short chain fatty acids—acetate, butyrate, isobutyrate, propionate, formate, succinate; and amines—putrescine, cadaverine, trimethylamine produced by the anaerobes [8, 16, 25, 26]. The anaerobes also utilise lactic acid as energy source to further propagate their survival and dominance [8, 25, 27].

Because there is no clear evidence of direct heterosexual transmission of BV-associated bacteria, BV is not usually described as a STI. Instead, due to its positive relationship with frequent unprotected sexual intercourse with new and multiple sexual partners, it has been termed a sexually enhanced infection [24, 27]. However, the reduction in the incidence of BV with the use of condom, the detection of BV-associated bacteria in the male genital microbiota [28] and relapse after treatment if the woman continues to have unprotected sexual intercourse with the same sex partner are supportive of BV being sexually transmitted [27]. More recently, the spread of dispersed cells or cell aggregates from biofilms between hosts have boosted the putative STI profile of BV [29–33].

BV is diagnosed clinically by the Amsel’s criteria, and microbiologically by the Nugent scoring system [1, 27]. Both strategies do not detect polymorphonuclear leukocytes [34] in vaginal fluid due to the absence of an obvious inflammatory response [27]. The absence of an overt inflammatory response has been attributed to the immunomodulatory actions of some dysbiosis-associated metabolites such as acetate and succinate [25]. There is also a coevolution-determined immune tolerance between the gut-derived normal and abnormal vaginal microbiota [28].

The lack of clear signs of inflammation (immune response) against the pathogenic bacteria has encouraged the description of BV as a microbiological and immunological conundrum. Hence, we conceptualised this review to examine the plausible mechanisms undergirding immune suppression and evasion in bacterial vaginosis. The composition of cervicovaginal mucosal barrier; degradation of the mucosal barrier and immunoglobulin A (IgA); *G. vaginalis* haemolysin (vaginolysin); diminished IgA response against vaginolysin; mucosal sialic acid degradation, foraging and overall depletion; inhibition of IL-8-induced neutrophilic infiltration and metabolite-induced incapacitation of neutrophil and monocyte chemotaxis were discussed. We also considered the tolerance/resistance to both host and microbial antimicrobial molecules mounted by BV-associated biofilms. A plausible role of sialic acid-binding immunoglobulin-like lectins (SIGLECS) and therapeutic value of sialidase inhibition were also accentuated.

We evaluated the hypothesis that sialidase, supported by immunomodulatory metabolites all produced by BV-associated bacteria, is central to the immunosuppression observed in BV that accounts for a high rate of relapse/recurrence, which appear to underpin the strong association between BV and spontaneous preterm birth.

### Cervicovaginal Mucosal Barrier Composition

**Fig. 1:**
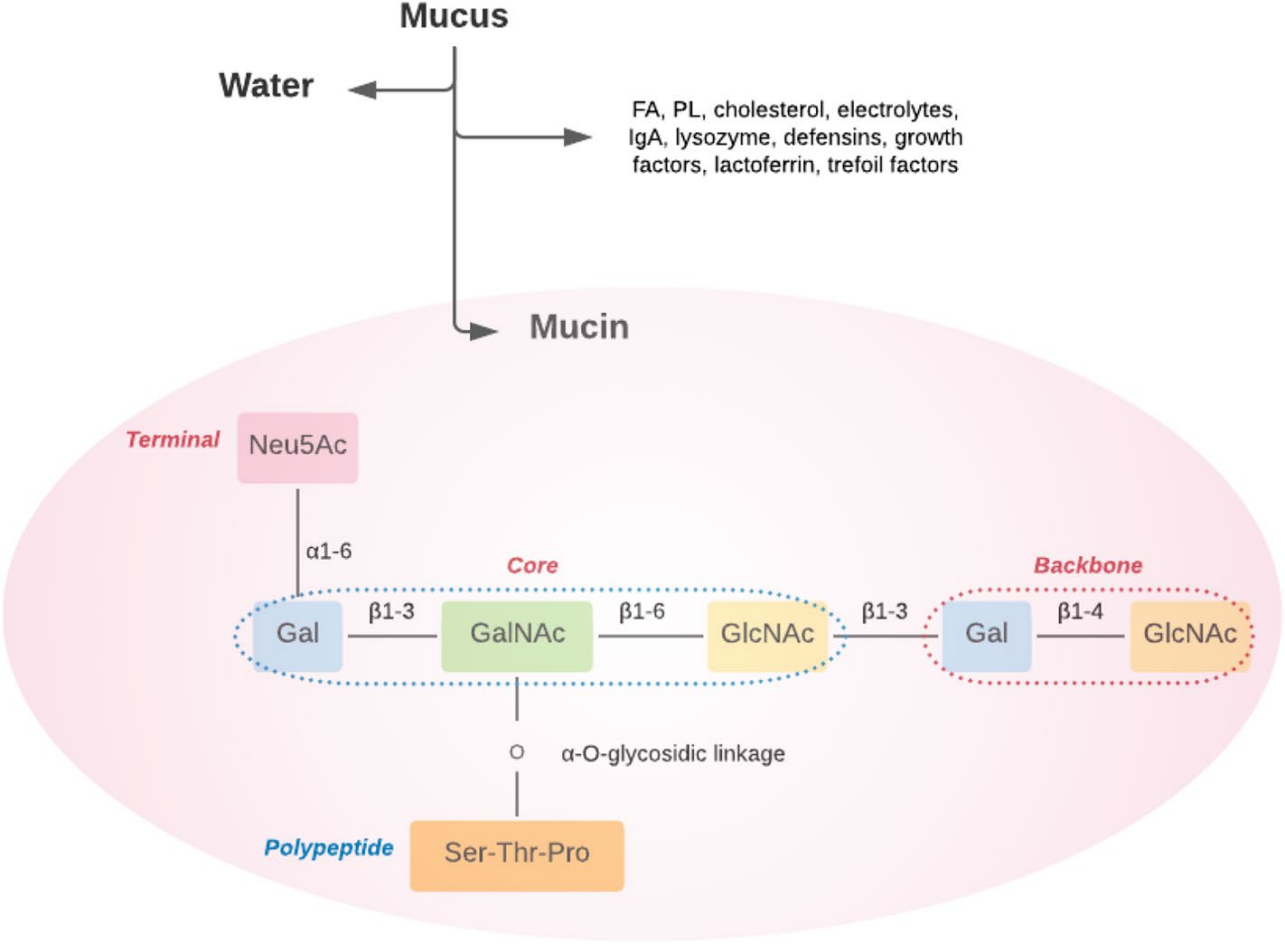
Biochemical composition of mucus and the structure of mucin glycoprotein showing the core, backbone and terminal domains. Mucins are highly glycosylated with carbohydrates making up ~ 85% of their dry weight and protecting them from proteolysis [37]. *FA*, fatty acid; *Gal*, galactose; *GalNAc*, *N*-acetylgalactosamine; *GlcNAc*, *N*-acetylglucosamine; *IgA*, immunoglobulin A; *Neu5Ac*, *N*-acetylneuraminic acid (sialic acid); *PL*, phospholipid; *Pro*, proline; *Ser*, serine; *Thr*, threonine

The mucus lining of the cervicovaginal epithelium is composed mainly of water (~ 95%) and mucin that accounts for its viscoelastic gel-like properties [36, 37]. Mucus also contains fatty acids, phospholipids, cholesterol, electrolytes and proteins with non-specific antimicrobial actions including immunoglobulin A, lysozyme, defensins, growth factors, lactoferrin and trefoil factors (Fig. 1) [36–38]. Cervicovaginal mucus provides a thick physical protective barrier and lubrication that prevents contact between epithelial cells and pathogens [39].

Mucins are large extracellular glycoproteins that are linked by α-O-glycosidic linkages [36, 37]. The terminal carbohydrate side chains protect the central protein core from degradation by proteolytic enzymes. The terminal side chains comprise mainly of sialic acid, L-fucose and sulphates, whilst the backbone contains galactose and *N*-acetylglucosamine. The inner protein core consists of polypeptides with multiple tandem repeats of serine, threonine and proline residues (STP repeats) that constitute > 60% of the amino acids; and cysteine-rich regions located at the amino and carboxy terminals and occasionally intermixed between the STP repeats. The O-glycosidic linkage to the STP repeats of the protein core is through *N*-acetylgalactosamine [36–38], whilst the cysteine-rich regions contain comparatively little glycosylation [36]. More details on current understanding of the interactions between cervicovaginal microbial communities, mucus barrier and cervical mucus plug in both physiological and BV states; as well as specific mucins and their role in BV can be found in the review by Lacroix et al. [40].

### Degradation of Cervicovaginal Mucosal Barrier and Immunoglobulin A

Mucins provide protection against pathogenic bacteria and fungi, and protects the upper genital tract from microbial invasion [37]. Sialic acid (*N*-acetylneuraminic acid, Neu5Ac) in mucin inhibits bacterial adhesion to vaginal epithelial cells and biofilm formation. Hydrolysis of sialic acid terminal on the glycans of mucous membranes by sialidase facilitates adhesion of bacteria on the vaginal epithelium and formation of biofilms [37, 41, 42]. Sialidase is produced by *G. vaginalis, Prevotella* spp., *Bacteroides fragilis, Mycoplasma hominis* and *Mobiluncus* spp. [37, 39].

In addition to sialidase, other bacterial glucosidases degrade various carbohydrate residues of mucins [37] and expose the inner protein core to proteolysis by proteases including prolidase (proline dipeptidase) produced by *G. vaginalis*, *Mobiluncus* spp. and *Peptostreptococcus* spp. Sialidase and other glucosidases also cleave the terminal sialic acid and catalyse proteolysis of sialoglycoproteins of fibronectins and cell adhesion molecules, that also participate in mucosal barrier and immune functions [4, 43, 44].

**Fig. 2:**
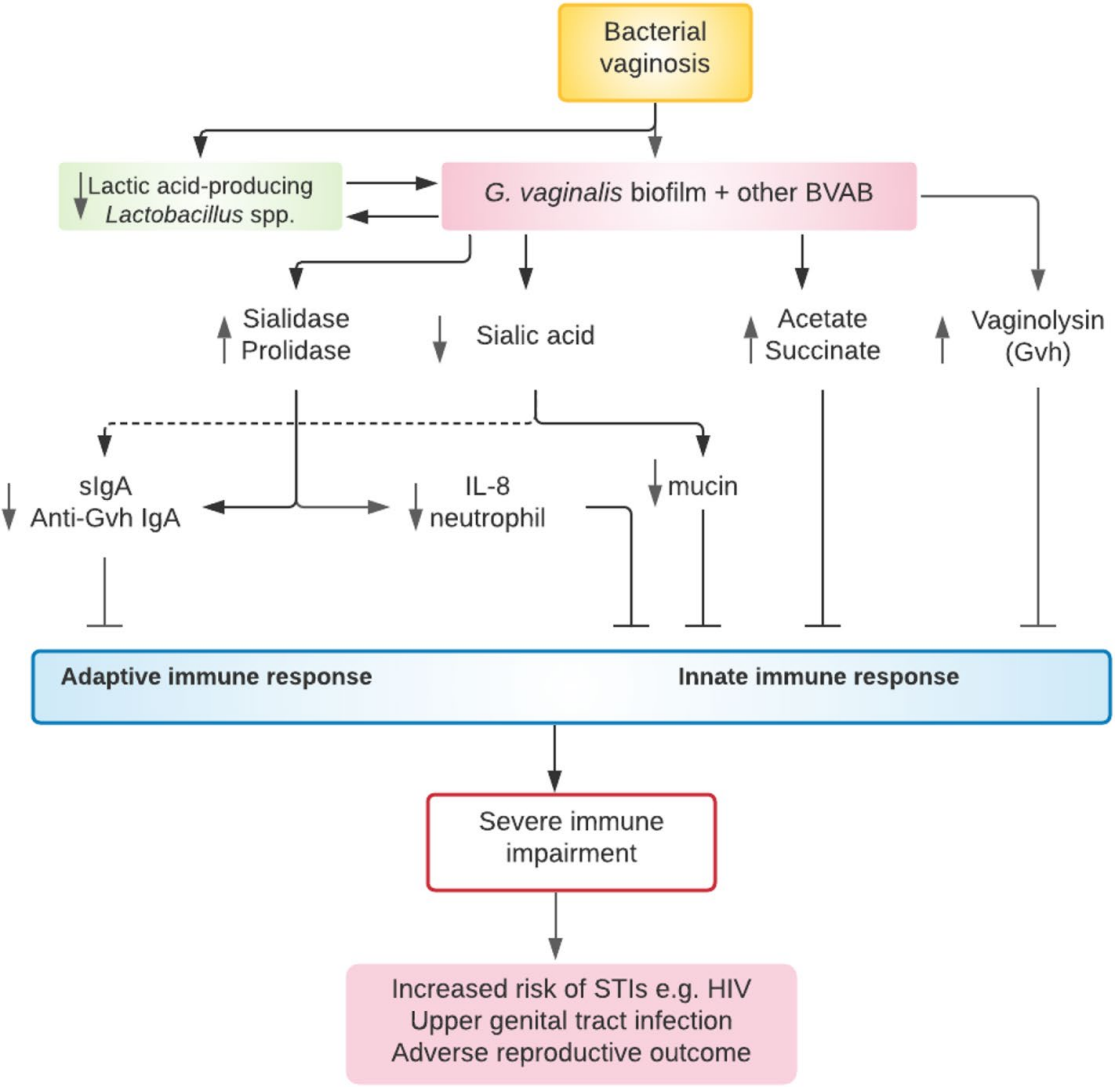
Plausible mechanisms of immune impairment and evasion in bacterial vaginosis infection. BV-associated bacteria suppress and overwhelm the host immune response by producing several virulent factors including mucolytic enzymes, cholesterol-dependent cytolytic toxins and short chain fatty acids that can act singly but often synergistically. *Anti-Gvh IgA*, immunoglobulin A against *Gardnerella vaginalis* haemolysin; *BVAB*, bacterial vaginosis-associated bacteria; *Gvh*, *Gardnerella vaginalis* haemolysin; *HIV*, human immunodeficiency virus; *IL*, interleukin; *sIgA*, secretory immunoglobulin A; *STI*, sexually transmitted infection

Sialidase also cleaves the terminal sialic acid from secretory IgA (sIgA) thereby increasing its susceptibility to proteolytic degradation [43, 45, 46]. The carbohydrate residues of sIgA are overlaid and protected by the terminal sialic acid residues. Removal of sialic acid exposes the carbohydrate residues to hydrolysis by exoglycosidases (galactosidase, glucosidase, hexosaminidase) [37, 43]. Desialylation or deglycosylation of the secretory component of sIgA hampers immune response by facilitating its proteolysis [43] by proteases produced by *G. vaginalis, Prevotella* spp. and *Ureaplasma urealyticum* [37]. The non-specific antibacterial activities of sIgA are eventually compromised (Fig. 2) [46].

Furthermore, other pathways of impaired immune response and evasion adopted by BV-associated bacteria include the enhanced activity of haemolysin (vaginolysin) secreted by *G. vaginalis* and ineffective IgA response against vaginolysin; the liberation and general depletion of mucosal sialic acid; the inhibition of cytokine-induced neutrophilic infiltration; and metabolite-induced paralysis of polymorphonuclear leukocyte chemotaxis. Other pathways also include the tolerance/resistance of BV biofilms mounted against both host defence and antimicrobial molecules (Fig. 2) and the putative action of SIGLECS are discussed in detail below.

### G. vaginalis Haemolysin (Vaginolysin) and Diminished IgA Response Against Vaginolysin

*G. vaginalis* also obliterates the vaginal epithelial barrier function by the formation of pores on the membranes and lyses of the cells. Disrupted membrane integrity leads to cell death and loss of cellular function [47]. This virulence mechanism is perpetrated by a *G. vaginali*s haemolysin (Gvh), which is a pore-forming cholesterol-dependent cytolysin (CDC) that is cytotoxic to eukaryotic cells including red blood cells [48, 49]. In line with CDC nomenclature, the 57 kDa toxic protein was later named vaginolysin (Fig. 2) [47, 50, 51].

Vaginolysin is cytotoxic to vaginal epithelia cells [41], in a species-specific manner that is dependent upon the recognition of human complement regulatory glycoprotein CD59 and membrane cholesterol [47, 50]. It is very active at high pH (5.0–7.5) as seen in BV with significantly lower levels observed when *Lactobacillus crispatus* dominates the microbiota [47]. Vaginolysin also induces protein kinase-dependent apoptosis in human epithelial and red blood cells [4, 50]. Through the destruction of the vaginal epithelial cells (desquamation), vaginolysin neutralises the physical and biochemical protection against pathogens mounted by the epithelial cells [41, 52].

In about 60% of women with BV, there is an adaptive antigen-specific immune response involving a vaginal mucosal IgA response against Gvh [23, 48, 49]. This mucosal adaptive immune response is diminished when sialidase and prolidase levels are elevated [53], with the attendant increased cleavage of vaginal fluid IgA (Fig. 2) [45]. Increased cleavage of IgA neutralises the anti-Gvh IgA protection against Gvh [53], and allows the haemolytic toxin to evade clearance and perform its cytolytic activity unabated leading to destruction and desquamation of the vaginal epithelial cells that eventually form the characteristic clue cells of BV infection [41, 49, 54]. Clue cells are formed when BV-associated bacteria such as *G. vaginalis, Bacteroides* spp. and *Mobiluncus* spp. are attached to exfoliated vaginal epithelial cells when lactobacilli are depleted and vaginal pH is greater than 4.5 [1, 55–57]. The attached bacteria release lytic enzymes such as sialidase and vaginolysin which facilitate invasion and destruction of the epithelial cells [39, 41, 58]. The host immune response is consequently suppressed [4]. High levels of anti-Gvh IgA appear protective against adverse pregnancy outcomes [23, 59]. BV-positive women with strong anti-Gvh IgA response and low sialidase and prolidase activities are not at risk of adverse pregnancy outcome such as low birth weight [60].

Taken together, the sialidase-induced deglycosylation and increased proteolysis of IgAs [43] as well as degradation of the protective mucus of vaginal epithelial barrier [4] attenuates the host IgA-mediated and innate immune responses. This possibly diminishes the ability of the female reproductive mucosa to neutralise and eliminate pathogens [43]. The integrity of the vaginal mucosal epithelium and cervical mucus is compromised, thereby facilitating ascending genital tract infection [22, 27, 39, 61].

Sialidase and β-N-acetyl-hexosaminidase are significantly increased in BV [3], and increased sialidase and/or prolidase activity is associated with preterm birth, premature rupture of membranes, low birth weight and very low birth weight [3, 4, 59–62] when combined with vaginal pH > 5 [45]. Sialidase predicted preterm delivery (≤ 34 weeks) with a high specificity and positive predictive value in women with BV or intermediate microflora [45].

### Sialic Acid Degradation, Foraging and Depletion

**Fig. 3:**
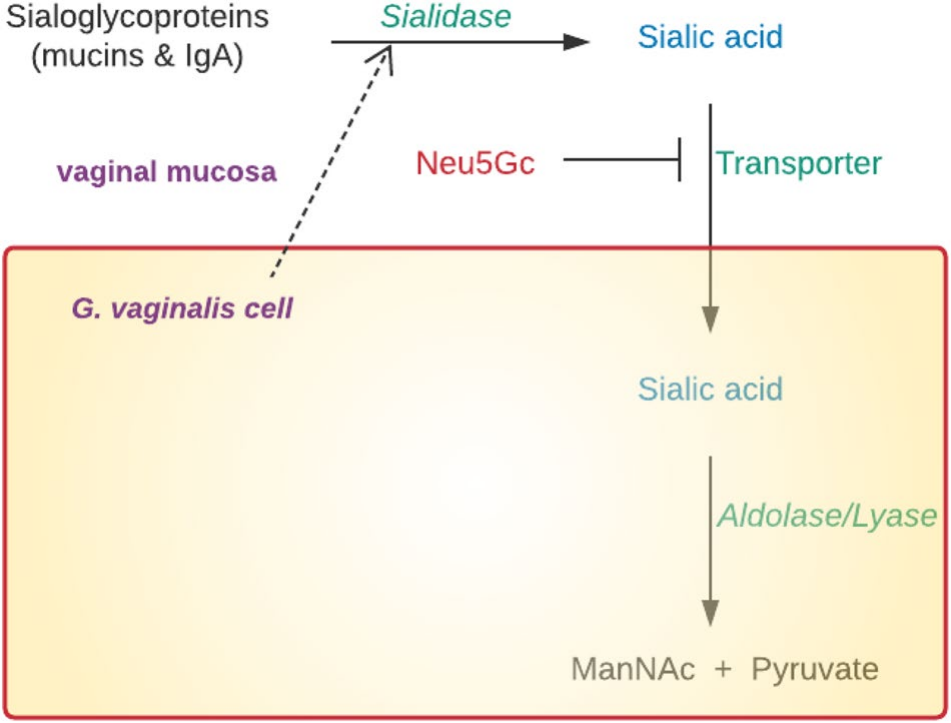
Sialic acid degradation, foraging and depletion induced by *G. vaginalis*. Sialic acid is cleaved from mucosal sialoglycoproteins such as mucins and immunoglobulin A (IgA) that form the first line of defence against invading pathogens. The free extracellular sialic acid is then captured and ingested by *G. vaginalis* via a high-affinity transport system that can be inhibited by *N*-glycosylneuraminic acid (Neu5Gc). Intracellular sialic acid is eventually catabolised by aldolase/lyase to *N*-acetylmannosamine (ManNAc) and pyruvate. This dampens the host immune response against bacterial vaginosis-associated bacteria leading to the absence of an obvious inflammation and increased risk of adverse reproductive outcomes

Vaginal sialidase activity is diagnostic of BV and independently correlates with risk of ascending genital tract infections and preterm birth [39]. Sialic acid-rich components of mucus such as mucin and IgA have protective and immunological functions [39]. Glycosylated mucus proteins contain about 16% sialic acid by weight [39]. To further evade host immune response, *G. vaginalis* employs sialidase to degrade and deplete vaginal mucus components containing sialic acid. Extracellular sialidase hydrolyses mucosal sialoglycoproteins and the cleaved sialic acid is transported by a high-affinity transport system into the bacterium. This is an active process enhanced by glucose but inhibited by excess *N*-glycosylneuraminic acid (Neu5Gc). The ingested sialic acid is then catabolised by intracellular aldolase/lyase reaction to *N*-acetylmannosamine (ManNAc) and pyruvate (foraging) (Fig. 3) [39]. Sialic acid catabolism provides nutrients that support bacterial growth [63]. This sialidase activity, which is not observed in healthy controls, i.e. women with healthy lactobacilliary vaginal microbiota [39, 43], leads to significantly reduced vaginal sialic acid in women with BV [39].

Depletion of the components of the protective mucosal barrier facilitates microbial adhesion and invasion of the upper genital tract [1, 39]. Though the vaginal sialidase activity and sialic acid foraging have been characterised in *G. vaginalis* extensively, other BV-associated bacteria such as *Bacteroides* spp. and *Prevotella* spp. can also produce the same pathogenic effect [39]. Future studies can determine the association of vaginal bacterial composition, level of sialidase activity and degree of sialic acid depletion in pregnant and non-pregnant women. The possibility of these variables singly or in combination to distinguish women at risk of preterm birth or other adverse reproductive outcomes can also be investigated.

### Inhibition of IL-8-Induced Neutrophilic Infiltration

The production of sialidase and prolidase by BV-associated bacteria is associated with increased cervicovaginal IL-1β. In spite of the increase in IL-1β levels, there is no concomitant increase in the neutrophil chemoattractant and activator, IL-8 [23, 64]. Though IL-1β promotes IL-8 secretion, IL-8 concentration and vaginal neutrophils are low in women with BV [64, 65]. The bacterial hydrolytic enzymes downregulates the IL-8/IL-1β ratio thereby blunting the IL-1β-induced proinflammatory cascade leading to reduction in neutrophil infiltration typical of BV [64].

IL-8 levels were inversely correlated with elevated sialidase and prolidase, and positively associated with anti-Gvh IgA response and neutrophils in women with BV [23, 53, 64], i.e. both innate and adaptive immune responses are impaired in BV [44]. In vivo experiments have shown that BV-associated bacteria can circumvent immune response by inhibiting IL-8 production and stability or secrete factors that facilitate IL-8 degradation [2]. The obvious decrease in vaginal neutrophils is the hallmark of BV, which indicates suppressed innate mucosal immunity (Fig. 2) [65].

Sialidase and prolidase can also degrade cationic antimicrobial polypeptides (cAMPs) such as human β defensins 1 and 2, secretory leukocyte protease inhibitor (SLPI) [64]; lactoferrin, some cytokines and cellular receptors such as Toll-like receptors (TLR) 2 and 4 [23, 66], which are the main effector molecules of vaginal innate and adaptive mucosal immunity against pathogens [1, 65]. The cAMPs are also engage in protection against the proteolytic enzymes released by pathogenic bacteria, fungi and some viruses as well as direct microbial killing [52]. Therefore, degradation of these protective molecules impairs the immune response and may increase the risk of poor reproductive outcomes in pregnant women with BV [64].

### Incapacitation of Neutrophil and Monocyte Chemotaxis

Neutrophil and monocyte chemotaxis is vital to a potent innate immune response to pathogenic stimuli. BV-associated bacteria metabolise carbon sources in the vaginal environment to short chain volatile fatty acids (SCFAs) including acetate, butyrate, isobutyrate, propionate and the non-volatile fatty acid–succinate. These metabolites increase vaginal pH that permits the colonisation of the cervicovaginal space by BV-associated bacteria and predispose the host to infection [1]. In addition to this already deleterious phonotype, the metabolites especially acetate and succinate are capable of inhibiting chemotaxis of immunocompetent cells including neutrophils and monocytes [67, 68], thereby incapacitating the host response against invading pathogens, and promoting infection and adverse reproductive outcomes such as preterm birth [25] (Fig. 2). For instance, succinate inhibits phagocytosis of *E. coli* as well as neutrophil chemotaxis. This anti-chemotactic action helps the infectious agents to dodge phagocytosis and the resultant neutrophil activity, and could account for the absence of pus cells (polymorphonuclear leukocytes) in vaginal secretions of women with BV [67]. The evasion of phagocytosis is usually the first action by pathogens in the induction of any infection including purulent (pyogenic) infections. The next action is prolong inhibition of neutrophil and monocyte chemotaxis which permits growth, proliferation and colonisation, and infection is established [67].

Succinate appears to be more potent than acetate in chemotaxis inhibition. However, both metabolites may act synergistically to amplify the anti-chemotaxis observed in BV. Lactic acid that is predominant in the cervicovaginal fluid of healthy women does not exhibit this inhibitory effect. In vitro experiments have shown that supernatants of *Prevotella* spp. and *Mobiluncus* spp. produced significantly higher succinate and acetate, thus, more anti-chemotatic effect compared to *Gardnerella* spp. Succinate and acetate are believed to disrupt the binding of chemotactic factor to the surface of polymorphonuclear leukocytes. Some *Prevotella* spp. have also been reported to produce succinate and acetate that inhibit chemotaxis of leukocytes irreversibly [67].

Because BV is a polymicrobial dysbiotic condition, the mixed microbiota could act in synergy, i.e. each organism amplifying or potentiating the virulence mechanism of the other, thereby contributing to the overall evasion of host immune response [67]. For instance, *Prevotella* spp. and *Mobiluncus* spp. could produce more succinate and acetate to incapacitate neutrophils [67] allowing *G. vaginalis* ample time to proliferate and form biofilms that are resistant to the antimicrobial and anti-inflammatory effects of lactic acid-producing lactobacilli, mucosal defences and antibiotics. The *G. vaginalis* biofilm in turn provide a scaffold for the build-up of other BV-associated bacteria [41, 69]. This produces a more formidable resistance in addition to evading the host immune response resulting in the persistence and recurrence of BV. This synergistic biofilm formation effect between BV-associated bacteria can also be achieved via sialidase and vaginolysin-mediated mucosal/innate defence impairment [41]. The polymicrobial synergistic interaction linked to the pathomechanism of BV is established as *G. vaginalis* is present in nearly 90% of women without BV and as such may not be the sole causative organism in all instances [70].

### Polymicrobial Biofilm Formation

A biofilm is a community of microorganisms encapsulated in a polymeric matrix of polysaccharides, proteins and nucleic acids, and attached to a surface [71]. The stages of biofilm formation include the following: (i) adhesion, (ii) microcolony formation and coaggregation, (iii) maturation and (iv) dispersion (erosion and sloughing induced by hydrolytic enzymes or increased pH in the vagina) [7]. Bacteria within biofilms are usually shielded from the host immune response and antibiotic therapy resulting in the persistence of such infections. Standard antibiotics such as metronidazole are unable to completely clear BV vaginal biofilms-associated bacteria [72]. For example, oral metronidazole therapy on adherent *G. vaginalis* biofilms only temporarily suppressed the biofilms, which immediately resumed pathogenic activity after treatment cessation [73]. BV biofilm-forming bacteria also exhibited resistance to metronidazole, tinidazole and clindamycin in an in vitro study [74]. In this study [74], *G. vaginalis* had the greatest virulence capacity indicated by higher initial adhesion and cytotoxicity of epithelial cells, as well as greater tendency to form a biofilm compared to other biofilm-forming BV-associated bacterial species including *Streptococcus agalactiae*, *Gemella haemolysans*, *Enterococcus faecalis*, *Propionibacterium acnes*, *Mycoplasma hominis* and *Escherichia coli*. [74]. Therefore, biofilm formation also accounts for the high rate of relapse and recurrence seen in BV cases [72–74]. More than 50% of women treated for BV will have recurrent episode(s) within 6–12 months [75]. That is, in addition to the overgrowth of anaerobes, BV is associated with the presence of a dense, structured and adherent polymicrobial biofilm assembled by *G. vaginalis* on the vaginal mucosa [76].

It is believed that the initiation and progression of BV is dependent/induced by the formation of *G. vaginalis* biofilm [72, 76], which can re-form following oral metronidazole treatment in some instances [73], as it serves as reservoir for regrowth of pathogens [77]. *G. vaginalis* also produces extracellular DNA (eDNA) that stimulates the formation of extracellular polymeric substance matrix implicated in biofilm maturation and persistence [7, 78]. This initial biofilm serves as the scaffold for the coaggregation of other BV-associated anaerobes such as *Atopobium vaginae, Prevotella bivia, Mobiluncus mulieris*, *Fusobacterium nucleatum* and *Peptoniphilus* spp. [7, 30, 41, 73, 76, 79–85], thereby forming an intractable virulent polymicrobial alliance attached tightly to the surface of the vaginal epithelium like a “brickwork”[76], and persistently expressing destructive immune suppressing molecules such as hydrolytic enzymes, vaginolysin [41, 72, 73] and SCFAs [1, 25]. Other BV-related species including *Bacteroides* spp., *Streptococcus* spp., *Veillonella* spp., [76], *E. coli, E. faecalis* and *Actinomyces neuii* [7, 41, 84, 86] have also been found on biofilms augmenting the growth of *G. vaginalis*. *G. vaginalis* serves an early coloniser in the development of multispecies biofilm, whilst the other BV-associated species serve as second/third colonisers [7]; although the process may be initiated by *Peptoniphilus* spp. [87] instead of *G. vaginalis*. The extracellular polymeric matrix produced by the adherent bacterial species encapsulates and shields the biofilm from host immune system and antibiotics [72].

By releasing sialic acid from mucosal sialoglycans, sialidase unmasks the cryptic host ligands required for bacterial adherence. This enhances biofilm formation, bacterial colonisation [37, 41, 42, 63], loss of membrane integrity and damage to epithelial cells [47, 88]. The cytotoxic action of vaginolysin also contributes to vaginal epithelial cell desquamation that manifest as the characteristic clue cells of BV [39, 41, 55, 57, 58]. *A. neuii* or *E. faecalis* when colonising biofilms formed by *G. vaginalis* can stimulate overexpression of vaginolysin and sialidase genes by *G. vaginalis* cells to facilitate formation of clue cells [41]. By contrast, health-promoting *L. crispatus* represses vaginolysin expression by *G. vaginalis* and reduces vaginal epithelial cell cytotoxity [47, 89]. However, *G. vaginalis* still adheres to vaginal epithelial cells in the presence of *L. crispatus* with the assistance of *Lactobacillus iners* [82, 90] and *Peptoniphilus* spp. [79, 82, 87]. Biofilms play vital roles in the pathogenesis of BV mounting tolerance or resistance to the host-microbial defence mechanisms of normal vaginal microbiome including lactic acid, H_2_O_2_, mucosal immune defences, antibodies, as well as demonstrating enhanced antibiotic tolerance [4, 41, 72, 73, 91].

BV-associated pathogens also produce other substances that target B-cells and immunoglobulins such as haemolysin III, superantigen (L-protein) and peptostreptococcal albumin-binding proteins [4], and suppress the host immunity. When these BV-associated organisms form a biofilm with each demonstrating its virulence as well as immune suppressing or subversion properties, there is rarely a noticeable inflammatory response. Hence, established but uncomplicated BV is typically characterised by lack of leukocytes on microscopy, the vagina is not inflamed or reddish, and no significant burning sensation, pain or dyspareunia is observed [34].

### SIGLEC-Induced Negative Regulation of Immune Response

The lipopolysaccharide (LPS) receptor complex comprise of pattern recognition receptors (PRRs) that are sialylated glycoconjugates, i.e. TLR4, myeloid differentiation protein 2 (MD-2) and CD14. CD14 is a glycosylphosphatidylinositol-anchored glycoprotein expressed on leukocytes, which facilitates binding of LPS to TLR4. LPS/TLR4 binding initiates NF-κB-mediated proinflammatory cytokine production [92]. Elevated sialidase activity as seen during immune cell differentiation/activation [93] and BV [3], cleaves sialic acid from PRRs on the surface of immune cells [23, 66, 93]. Sialic acid removal facilitates response of the PRRs to LPS and production of proinflammatory cytokines [93].

However, inhibitory receptors such as sialic acid-binding immunoglobulin-like lectins (SIGLECS) expressed by immune cells attenuate the expected immune reaction by binding to the sialylated glycoconjugates (PRRs) [94]. For instance, CD33 (Siglec-3), the smallest member of the SIGLEC family, down-regulates TLR4 mediated signalling by binding to its *cis* ligand CD14. The CD14/CD33 interaction regulates the presentation of LPS from CD14 to TLR4, thereby down-regulating the LPS-NF-κB proinflammatory pathway [92]. It is plausible that this SIGLEC-mediated negative regulation of TLR4 signalling may contribute to the attenuation of pro-inflammatory response observed in BV. This warrants further investigation.

## Future Perspectives

The lack of an appropriate inflammatory response in BV despite obvious colonisation of the lower genital tract by pathogenic bacteria resulting from disruption of vaginal homeostasis has been a great concern to clinicians and researchers. This has greatly impacted early diagnosis and prompt treatment of women with the condition. Hence, recurrent BV remains a major public health burden especially in the pregnant population.

The molecular mechanisms of immune evasion in BV have been studied in isolation leading to absence of a comprehensive understanding and report of the factors that distinguish BV from vaginitis and other female genital tract infections. This is a critical review that attempts to discuss and link the probable mechanistic characteristics underpinning the enigmatic and precarious suppression and subversion of host immune system/response by BV-associated bacteria.

We observe that BV incorporates a spectrum of alterations including increased species biodiversity, production of immunomodulatory enzymes and metabolites, and suboptimal host biochemical and immunological responses [23, 45]. That is, it is not only a decrease in lactic acid-producing *Lactobacillus* spp. and overgrowth of mixed anaerobes, but also increase in mucin-degrading enzymes (e.g. sialidase and prolidase), acetate and succinate, and cytotoxins such as vaginolysin that characterise BV. This deleterious phenotype is aggravated when the organisms form biofilms that confer additional resistance to host defence and antibiotic clearance. The host response is characterised by reduced IL-8 levels and neutrophil infiltration. Therefore, the host-microbial interactions at the vaginal mucosa is pivotal to the sequelae of BV infection.

Though the underlying mechanistic factors can act synergistically, the impact of each of them differ amongst patients, and this is crucial for accurate diagnosis and tailored therapeutic interventions to preclude relapse and recurrence. Of these factors, sialidase appears to be central to the mechanistic pathways and has attracted more research attention, being employed as a diagnostic test for BV (BVBlue test) [95, 96]. Sialidase degrades sialic acid and by extension disrupts mucosal epithelial membranes and facilitates bacterial attachment, biofilm formation and IgA degradation. This sets the stage for the continuous production and activities of other virulence factors including vaginolysin and SCFAs. Elevated levels of sialidase are associated with preterm birth and low birth weight, with modest predictive capacity for preterm birth [3, 4, 59–62]. Elevated acetate levels have also predicted preterm birth especially in women presenting with symptoms of preterm labour [97–99]. Whether sialidase alone or in combination with other microbial virulence and/or host immune factors is clinically useful for the diagnosis of preterm birth and related poor pregnancy outcomes remains to be established and therefore necessitates further investigation.

Because of the immunosuppression and subversion of the immune response, BV women show highly variable clinical prognosis. Some BV infected women recover without treatment, some harbour the infection without symptoms for years especially when poorly treated, others recover after a single treatment, whilst others require multiple or continuous treatments. Consequently, women with BV can be subclassified into those that maintain local immune response and those with impaired immune response (Table 2) [46, 54]. BV-positive women that maintain local immune response show less aggressive disease and better prognosis. In contrast, those with compromised immune response induced mostly by high sialidase exhibit a more aggressive disease and are at greater risk of relapse and recurrence even though they are often asymptomatic. This sub-classification should improve treatment of BV-positive women [23].

**Table 2:** Immune classification of women with bacterial vaginosis

Low risk Maintained local immune response	High risk Impaired immune response	References
Low cleavage of IgA	Increased cleavage of IgA	[23, 46, 54]
Low sialidase and prolidase activity	High sialidase and prolidase activity	[23, 46, 54]
Positive anti-Gvh IgA response	No anti-Gvh IgA response	[23, 46, 54]
Increased IL-8 and neutrophil chemotaxis	Reduced IL-8 and neutrophil chemotaxis	[23, 46, 54]

*Anti-Gvh IgA*, immunoglobulin A against *Gardnerella vaginalis* haemolysin; *Gvh*, *Gardnerella vaginalis* haemolysin

Furthermore, the possibility of targeting (inhibiting) sialidase activity for therapeutic purposes can rehabilitate the dysbiotic vaginal microbiota to a lactobacilliary (eubiotic) status by suppressing sialidase-positive BV bacteria. As demonstrated in mice models, this would reduce the level of free sialic acid and starve BV pathogens such as group B *Streptococcus* of key nutrients, and prevent ascending genital tract infection and chorioamnionitis [88]. The anti-influenza drug Zanamivir has shown ability to inhibit sialidase activity and invasion of human cells by *G. vaginalis*. This can reduce bacterial adhesion and inflammation by maintaining functional IgA [43]. Some of our colleagues (Galleh et al.) [100] have also explored the effectiveness of plant-derived (e.g. Epicatechin gallate and Berberine chloride) and synthetic (2,3-Difluorosialic acid analogues) sialidase inhibitors compared to Zanamivir in the oral cavity. Further testing of the anti-sialidase activity of Zanamavir and the oral sialidase inhibitors in a polymicrobial vaginal microbiota model is imperative.

## Conclusion

In conclusion, sialidase is central to the immunosuppression observed in BV that accounts for the absence of an overt inflammatory response, many asymptomatic cases and high rate of relapse and recurrence. Its pathogenic effects are supported by other hydrolytic enzymes, haemolytic toxins and immunomodulatory cytokines and metabolites. All these factors are produced or induced by several BV-associated bacteria with *G. vaginalis* as the prime culprit in most cases. Although sialidase and acetate have shown capacity to identify women at risk of preterm birth, further studies are required to validate their clinical utility independently or combined with the other virulence factors. Such studies can be conducted in women of different ethno-racial groups and/or living in different geographic locations. Finally, if the natural and/or synthetic sialidase inhibitors are effective as therapeutic (eubiotic) agents in the vaginal milieu, they could potentially reduce the incidence of recurrent BV in the general and pregnant populations as well as the excessive use of antibiotics and antibiotic resistance.

## Data Availability

Not applicable.
